# Recent Advances and Future Directions in the Understanding of Mucormycosis

**DOI:** 10.3389/fcimb.2022.850581

**Published:** 2022-02-24

**Authors:** Victoriano Garre

**Affiliations:** Department of Genetics and Microbiology, Faculty of Biology, University of Murcia, Murcia, Spain

**Keywords:** CRISPR-Cas, diagnosis, antifungal resistance, phagocytosis, infection, macrophages

## Abstract

Mucormycosis is an emerging infection caused by fungi of the order Mucorales that has recently gained public relevance due to the high incidence among COVID-19 patients in some countries. The reduced knowledge about Mucorales pathogenesis is due, in large part, to the historically low interest for these fungi fostered by their reluctance to be genetically manipulated. The recent introduction of more tractable genetic models together with an increasing number of available whole genome sequences and genomic analyses have improved our understanding of Mucorales biology and mucormycosis in the last ten years. This review summarizes the most significant advances in diagnosis, understanding of the innate and acquired resistance to antifungals, identification of new virulence factors and molecular mechanisms involved in the infection. The increased awareness about the disease and the recent successful genetic manipulation of previous intractable fungal models using CRISPR-Cas9 technology are expected to fuel the characterization of Mucorales pathogenesis, facilitating the development of effective treatments to fight this deadly infection.

## Introduction

Mucormycosis is a deadly agioinvasive infection caused by fungi of the order Mucorales with an incidence that has grown in the last years (Prakash and Chakrabarti, 2019; Skiada et al., 2020), although this number is likely to be severely underestimated (Skiada et al., 2020; Soare et al., 2020). Traditionally, this infection has received scarce attention because of the low number of cases in comparison with other more frequent fungal infections, but the emerge of the SARS-CoV-2 disease (COVID-19) pandemic has increased the incidence of fungal infections, rising the concern about their risks (Ghosh et al., 2021). The upsurge in COVID-19-associated mucormycosis with a prevalence 50 times higher than the uppermost recorded data (Hussain et al., 2021) has highlighted the unmet need to better understand mucormycosis (Stone et al., 2021).

Mucormycosis is a complex fungal infection for several reasons. Despite affecting most frequently individuals with underlying pathologies reducing the immune response, around 19% are immunocompetent patients that have suffered trauma or burn wounds (Roden et al., 2005; Jeong et al., 2019b). In addition, the clinical presentation is diverse and linked to the underlying pathology with rhino-orbital-cerebral mucormycosis as the most frequent manifestation followed by cutaneous, pulmonary, disseminated, gastrointestinal, and others (Jeong et al., 2019b). Finally, it is caused by 38 different species corresponding to 11 genera, being *Rhizopus* the most frequent genus, followed by *Mucor* and *Lichtheimia* (Roden et al., 2005; Walther et al., 2019). Most knowledge about mucormycosis derived from these three genera, but fully understanding of the disease requires more work studying unusual Mucorales infections (Gomes et al., 2011).

The disease shows high mortality, and its reduction is confronted with several problems. The first one is the scarce tests available for the diagnosis in the early stages of the infection (Skiada et al., 2018; Skiada et al., 2020; Lackner et al., 2021). Thus, preemptive therapy for patients at a high risk for mucormycosis has been proposed as a strategy to reduce mortality (Chamilos et al., 2008). Once a diagnosis is made, management is challenging because treatment options are reduced, with surgery and amphotericin B therapy being the most extended and effective options. The actual problem is evidenced by the fact that mortality has not improved significantly in recent years despite the advent of newer antifungals (Skiada et al., 2018; Jeong et al., 2019a). The development of new efficient treatments faces the problem of poor knowledge about the Mucorales physiology, including molecular mechanisms that govern pathogenesis, which is motivated, in large part, by their genetic intractability (Soare et al., 2020). This review recapitulates the latest advances in the knowledge of Mucorales pathology aiming to highlight the major accomplishments needed to reduce the impact of mucormycosis on human health.

## Development of Rapid and Specific Diagnosis Procedures Is an Urgent Need

Appropriate and timely treatment of Mucormycosis to reduce mortality rate requires early detection of the pathogen, which is one of the main targets of current research. Diagnosis is still being performed by classical methods comprising histology/microscopy and culture that show low sensitivity and need a long time, respectively (Skiada et al., 2018; Skiada et al., 2020; Lackner et al., 2021). Moreover, morphological identification is not reliable and molecular identification is strongly recommended (Sagatova et al., 2016). Despite internal transcribed spacer (ITS) sequencing is strongly supported, matrix assisted laser desorption ionization time of flight (MALDI-TOF) has been proved to be a reliable and rapid method for the identification at species level, although it relies on in-house databases (Schwarz et al., 2019). Consequently, an intense effort is being devoted to developing methods based on the detection of molecules that allow the rapid and specific identification of the pathogen. Many approaches have been successful in the identification of Mucorales by analyzing DNA from either tissue samples or circulating DNA, also called cell-free DNA (cfDNA), in blood and even urine using PCR-based methods (Millon et al., 2016; Baldin et al., 2018). High performance of Mucorales PCR in blood samples comes from the high cfDNA levels, which are probably caused by the fact that Mucorales are much more angioinvasive than other fungi (Millon et al., 2016). In addition, these methods are non-invasive and can provide earlier and rapid diagnosis, making them ideal. The main drawbacks are the lack of standardization and clinical evaluation. However, some initiatives are stimulating such as a multicenter study that showed good reproducibility and performance of quantitative PCR assays in serum samples, supporting the use of this technique as part of the diagnostic strategy for mucormycosis (Rocchi et al., 2021).

We should also follow up on the promising evolution of techniques with high sensitivity that analyze cfDNA in blood such as the already used whole genome sequencing (WGS) and forthcoming techniques based on CRISPR-Cas. WGS has been proved to be useful for molecular typing in outbreaks (Walther et al., 2020) and its application for early diagnosis using cfDNA in blood is promising (Shi et al., 2021). Several CRISPR-Cas-based point-of-care tests are in development or available to detect different viruses, which include a Cas nuclease coupled in most cases with detection by a lateral flow device (Ganbaatar and Liu, 2021). Despite they have not yet applied to fungal infections, their introduction could enhance the diagnosis of mucormycosis (Morio et al., 2020). There are additional incipient technical developments relying on the identify other molecules that all need further investigations. These comprise the use of serologic tests based on the recognition of fucomannan (Burnham-Marusich et al., 2018) and the analysis of breath volatile metabolites by thermal desorption gas chromatography/tandem mass spectrometry (Koshy et al., 2017).

## Mucormycosis Is Not One Disease, but Many

Most fungal infections are defined by the genus of the causative species, but mucormycosis is caused by 38 different species corresponding to 11 genera (Walther et al., 2019), an intricate situation since some pathogenic mechanisms could be species-specific. Pursuing an ideal treatment to combat the disease would require studying the pathogenesis in more than one genus to find common molecular mechanisms to all pathogenic Mucorales. On top of that, intraspecific genetic variation is presumably very high in Mucorales, but few, if any, comprehensive analyses have been performed for its evaluation. The possibility of performing WGS affordably has provided some hints about this genetic variation. Thus, genome length of *Rhizopus* species is highly variable, especially within *Rhizopus microsporus* (Gryganskyi et al., 2018). In addition, sequencing of 72 isolates from patients and hospital and regional environments revealed a remarkable genetic diversity within species, even affecting the number of putative genes (Nguyen et al., 2020). This variability may have a profound effect on characteristics linked to pathogenesis and resistance to antifungals, explaining why different clinical isolates of a species show a wide range of resistance to different antifungals (Borman et al., 2017). Therefore, we urgently need pan-genomic analyses in Mucorales similar to that performed in *Aspergillus fumigatus* (Barber et al., 2021) to discover the intraspecific genetic variation associated with human infection. In fact, the *A. fumigatus* study points out that a single or a small number of reference genomes do not necessarily represent the species as a whole (Barber et al., 2021).

## Mucorales Resistance to Antifungals

Mucorales show an intrinsic resistance to most antifungals used in clinic, except for amphotericin B, leaving clinicians with insufficient treatment options. This innate drug resistance could be linked to the widespread duplications of genome regions and whole genome in Mucorales (Ma et al., 2009), which also could affect pathogenesis. However, a few works have shed some light on the mechanisms that could explain this resistance. In one pivotal work, sequence alignment of the lanosterol 14α-demethylase CYP51 F5 proteins, the target for azoles, from several species revealed a conserved single amino acid substitution Y129F previously related to the resistance to short-tailed triazoles (Sagatova et al., 2016), suggesting that this change mediates the innate resistant of Mucorales to short-tailed triazoles (Caramalho et al., 2017).

Another common mechanism of acquired azole resistance involves the upregulation of multidrug transporters (Cowen et al., 2015; Revie et al., 2018). Deletion of two out of eight *M. lusitanicus* genes encoding putative ABC transporters of the pleiotropic drug resistance transporter subfamily only increased slightly the susceptibility to some azoles (Nagy et al., 2021). This small effect in antifungal sensibility could be in part explained by the upregulation of other transporter subfamily members, suggesting that the regulation of these genes are interconnected (Nagy et al., 2021).

In addition to the intrinsic resistance mechanisms described above, an epigenetic acquired mechanism has been described in *M. lusitanicus* that confers transient resistance. This mechanism generates RNA interference (RNAi)-dependent epimutants in response to the presence of a drug by silencing the gene encoding either the target protein (Calo et al., 2014) or the enzymes that convert the drug into a toxic compound (Chang et al., 2019). Interestingly, murine infection of *Mucor* led to increased rates of epimutation when isolates recovered from organs were exposed to the antifungal agent FK506, suggesting that growth in animal tissues could prime the rapid response of this mechanism to subsequent stresses, including antifungal exposure (Chang and Heitman, 2019). The ability of this epigenetic acquired resistance mechanism to target different genes suggests that it could contribute to the broad resistance of Mucorales to clinically available antifungal drugs (Chang et al., 2019). Despite these advances, additional work is required to understand the Mucorales mechanisms of antifungal resistance and their role *in vivo* infections due to the problems of correlating *in vitro* resistance with clinical outcome (Lamoth et al., 2021).

## Host Invasion and Damage

Mucormycosis is initiated by asexual resting spores that get in contact with an epithelium that prevents invasion and hence, epithelial damage may result in mucormycosis. Interestingly, a genetically modified larval zebrafish model reproducing epithelial damage has been developed that can be instrumental to understand fungal attachment and invasion. This model has revealed that loss of epithelial integrity promotes fungal adhesion and invasion mediated by exposure of extracellular matrix components, while the epidermal growth factor (EGF) signaling pathway provide a protective effect (Wurster et al., 2021). In addition, an intense research has been carried out to identify both the spore coat and host proteins that interact to facilitate the entry of the fungus. All invasive Mucorales species analyzed contain genes coding for spore coat proteins CotH and their copy number correlates with clinical prevalence and the ability to cause invasive disease (Chibucos et al., 2016). At least in *Rhizopus delemar*, different CotH proteins interact with different host receptors depending on the tissue. CotH3 interacts with glucose-regulated protein 78 (GRP78) on nasal epithelial and endothelial cells, whereas CotH7 recognizes integrin β1 on alveolar epithelial cells, which activates EGF signalling to induce fungal invasion of host cells (Gebremariam et al., 2014; Watkins et al., 2018; Alqarihi et al., 2020). This combination of interactions could explain that patients with diabetic ketoacidosis ( DKA) are more susceptible to rhinoorbital/cerebral disease because they overexpress both CotH3 and GRP78 (Alqarihi et al., 2020). 

Several works have suggested that Mucorales secrete proteins that could be toxic for the host cells. Aberrant vesicle trafficking in *M. lusitanicus* (Patiño-Medina et al., 2018; Patiño-Medina et al., 2019b) and tornadic shear stress in several Mucorales (Wurster et al., 2020) increase secretion of unknown proteins that result in higher virulence in animal models. However, the only toxin identified until now is mucoricin (Soliman et al., 2021), a protein secreted during the germination of *R. delemar* spores with structural and functional features similar to the plant toxin ricin that contributes to pathogenesis by enhancing angioinvasion, inflammation and tissue destruction. Importantly, the gene encoding for mucoricin is widely present in pathogenic Mucorales and hyphal extracts from some of them caused *in vitro* damage to human alveolar epithelial cells, suggesting that this toxin could be secreted by other Mucorales species (Soliman et al., 2021).

Understanding all the aspects of mucormycosis requires animal models that somehow simulate the comorbidities associated with the disease, although they rarely recapitulate clinical scenarios (Ben-Ami and Kontoyiannis, 2021). Despite the differences with humans, mouse is the preferred model to study infection because it is the best established laboratory mammal model with the same body temperature as humans (Jacobsen, 2019). Other vertebrates (rabbit and zebrafish) and alternative invertebrate hosts (*Drosophila melanogaster*, *Galleria mellonella*, and *Caenorhabditis elegans*), have been greatly contributed to characterize particular aspects of the disease. All models have advantages and disadvantages (Jacobsen, 2019) and the election depend on the answer to be addressed.

## Understanding the Fungal Response to the Macrophage Attack

In addition to the physical barriers, the immune effector cells play a central role in halting the progression of the infection. Professional phagocytes, including macrophages and neutrophils, are rapidly recruited to the infection point, but spores are phagocytized predominantly by macrophages (Andrianaki et al., 2018). However, macrophage-mediated phagocytosis fails to kill the Mucorales spores with apparent species-specific results. In *Rhizopus* species, phagocytosis prevents spore germination, but they remain viable for, at least, 10 days post-infection of immunocompetent mice (Waldorf et al., 1984; Andrianaki et al., 2018). On the contrary, in *Mucor* species, macrophages of a murine cell line are unable to block *in vitro* germination that results in macrophage death by lysis (Li et al., 2011) and apoptosis (Lee et al., 2015), also observed in an adult zebrafish infection model (López-Muñoz et al., 2018). In addition, both genera induce phagosome maturation arrest mediated by cell wall melanin and the calcineurin signal pathway in *Rhizopus* (Andrianaki et al., 2018) and *Mucor* species (Lee et al., 2015; Vellanki et al., 2020), respectively. Resting spores of *Lichtheimia corymbifera* also inhibits the intracellular acidification of macrophages *in vitro*, independently from melanin (Hassan et al., 2021). 

Despite the progress on Mucorales–macrophage interaction knowledge, more information is needed to understand the mechanisms used by Mucorales to survive inside the macrophage, which is supposed to facilitate dissemination. The advent of next generation sequencing and tractable genetic models, particularly *M. lusitanicus*, has informed about those regulatory mechanisms. Analysis of the interaction between *R. delemar* and macrophages *in vitro* revealed nutritional immunity *via* iron restriction inside the phagosome as an important host defense mechanism (Andrianaki et al., 2018) that provokes the induction of the majority of fungal genes implicated in iron acquisition and virulence (Ibrahim et al., 2010; Navarro-Mendoza et al., 2018). In addition, studying the response to phagocytosis in virulent and attenuated strains of *M. lusitanicus* discovered general and virulence-specific responses that probably are responsible for the metabolism shift to allow germination inside the phagosome and survival to macrophage attack, respectively (Pérez-Arques et al., 2019). Several genes were identified that play crucial role in germination inside the phagosome and virulence, including two encoding basic leucine zipper transcription factors (Atf1 and Atf2) that regulates germination at low pH, suggesting that an Atf-regulatory pathway is activated by the acidic environment of the phagosome (Pérez-Arques et al., 2019). Interestingly, most of the genes responding to phagocytosis, including *atf1* and *atf2*, are repressed by a non-canonical RNAi pathway (NCRIP) during saprophytic growth, suggesting that interaction of spores with macrophages inhibits NCRIP leading to the activation of the genetic program to overcome host defense mechanisms (Pérez-Arques et al., 2020). The transduction pathways that regulate the NCRIP and other pathogenic processes are unknown, but the identification of heterotrimeric G proteins involved in *M. lusitanicus* virulence suggests that we may be approaching to their identification (Patiño-Medina et al., 2019a; Valle‐Maldonado et al., 2020).

## New Genetic Tools to Study Gene Function

The last decade has produced a tremendous increase in the number of molecular techniques available to study the function of genes involved in pathogenesis. To date, *Mucor* is the Mucorales genus with the widest repertoire of genetic molecular tools to study gene function (Rodríguez-Frómeta et al., 2013; Binder et al., 2018; Nicolás et al., 2018; Vellanki et al., 2018; Navarro-Mendoza et al., 2019). Unfortunately, other relevant mucormycosis-causative genera lack comparable genetic toolbox and only the RNAi technology is available in *Rhizopus* (Ibrahim et al., 2010). Fortunately, this dismal situation is changing due to expansion of the CRISPR-Cas technology to medically relevant fungi (Morio et al., 2020). *M. lusitanicus* was again the first mucoralean species in which this technology was established and used for targeted mutation of several genes (Nagy et al., 2017). Similar approaches have been followed in *R. delemar* and *L. corymbifera*, but in these species, the procedures require optimization because they work exclusively in genes that produce a selectable phenotype after mutation (Bruni et al., 2019; Ibragimova et al., 2020), making unfeasible their widespread use. In this context, a promising plasmid-free CRISPR-Cas9-based procedure has been established for *R. microsporus* that produces stable transformants, allows targeted mutation of any gene, and uses microhomology repair templates, speeding up the whole process (Lax et al., 2021).

## Future Directions

The continuous increase in the mucormycosis incidence, further spurred by COVID-19 pandemic, could have a bright side if the number of research groups working in the field and funding also rise. This upsurge in cases could favor the standardization and clinical evaluation of molecular methods for an early diagnosis of the disease and the development of new treatments. In addition, research in promising immunotherapies against fungal ligand (CotH proteins) and mucoricin that are effective in laboratory assays could have further progress to reach the clinic (Gebremariam et al., 2019; Soliman et al., 2021). The development of these therapies and others are a major unmet need because investigational antifungals under testing have limited activity against Mucorales (Lamoth et al., 2022). It is expected that coming years will bring advances in the understanding of the molecular mechanisms controlling pathogenesis by improving the tractability of mucormycosis models by the hand of the CRISPR-Cas technology. Special attention should be also dedicated to deciphering the mechanisms that confer intrinsic and acquired resistance to antifungals. It is worth noting that the current situation hinders the development of treatments to combat the disease because the advances derived from one species lack detailed validation in other species or genera. Therefore, it is urgent to produce the tools allowing result verification in several species to identify Achilles heel disease that helps in reducing mortality of mucormycosis.

## Author Contributions

The author confirms being the sole contributor of this work and has approved it for publication.

## Funding

VG was funded by Fundación Séneca-Agencia de Ciencia y Tecnología de la Región de Murcia, Spain (20897/PI/18) and Agencia Estatal de Investigación (AEI), Spain (PGC2018-097452-B-I00, co-financed by FEDER).

## Conflict of Interest

The author declares that the research was conducted in the absence of any commercial or financial relationships that could be construed as a potential conflict of interest.

## Publisher’s Note

All claims expressed in this article are solely those of the authors and do not necessarily represent those of their affiliated organizations, or those of the publisher, the editors and the reviewers. Any product that may be evaluated in this article, or claim that may be made by its manufacturer, is not guaranteed or endorsed by the publisher.

## References

[B1] Alqarihi A. Gebremariam T. Gu Y. Swidergall M. Alkhazraji S. Soliman S. S. M.. (2020). GRP78 and Integrins Play Different Roles in Host Cell Invasion During Mucormycosis. MBio 11, e01087–e01020. doi: 10.1128/mBio.01087-20 32487760 PMC7267888

[B2] Andrianaki A. M. Kyrmizi I. Thanopoulou K. Baldin C. Drakos E. Soliman S. S. M.. (2018). Iron Restriction Inside Macrophages Regulates Pulmonary Host Defense Against *Rhizopus* Species. Nat. Commun. 9, 3333. doi: 10.1038/s41467-018-05820-2 30127354 PMC6102248

[B3] Baldin C. Soliman S. S. M. Jeon H. H. Alkhazraji S. Gebremariam T. Gu Y.. (2018). PCR-Based Approach Targeting Mucorales-Specific Gene Gamily for Diagnosis of Mucormycosis. J. Clin. Microbiol. 56, e00746–e00718. doi: 10.1128/JCM.00746-18 30068535 PMC6156309

[B4] Barber A. E. Sae-Ong T. Kang K. Seelbinder B. Li J. Walther G.. (2021). *Aspergillus fumigatus* Pan-Genome Analysis Identifies Genetic Variants Associated With Human Infection. Nat. Microbiol. 6, 1526–1536. doi: 10.1038/s41564-021-00993-x 34819642

[B5] Ben-Ami R. Kontoyiannis D. P. (2021). Resistance to Antifungal Drugs. Infect. Dis. Clin. North Am. 35, 279–311. doi: 10.1016/j.idc.2021.03.003 34016279

[B6] Binder U. Navarro-Mendoza M. I. Naschberger V. Bauer I. Nicolas F. E. Pallua J. D.. (2018). Generation of a *Mucor circinelloides* Reporter Strain—A Promising New Tool to Study Antifungal Drug Efficacy and Mucormycosis. Genes (Basel) 9, 613. doi: 10.3390/genes9120613 PMC6315630 30544643

[B7] Borman A. Fraser M. Palmer M. Szekely A. Houldsworth M. Patterson Z.. (2017). MIC Distributions and Evaluation of Fungicidal Activity for Amphotericin B, Atraconazole, Voriconazole, Posaconazole and Saspofungin and 20 Species of Pathogenic Filamentous Fungi Determined Using the CLSI Broth Microdilution Method. J. Fungi 3, 27. doi: 10.3390/jof3020027 PMC5715917 29371545

[B8] Bruni G. O. Zhong K. Lee S. C. Wang P. (2019). CRISPR-Cas9 Induces Point Mutation in the Mucormycosis Fungus *Rhizopus delemar* . Fungal Genet. Biol. 124, 1–7. doi: 10.1016/j.fgb.2018.12.002 30562583 PMC6784326

[B9] Burnham-Marusich A. R. Hubbard B. Kvam A. J. Gates-Hollingsworth M. Green H. R. Soukup E.. (2018). Conservation of Mannan Synthesis in Fungi of the Zygomycota and Ascomycota Reveals a Broad Diagnostic Target. mSphere 3, e00094–e00018. doi: 10.1128/mSphere.00094-18 29720523 PMC5932377

[B10] Calo S. Shertz-Wall C. Lee S. C. Bastidas R. J. Nicolás F. E. Granek J. A.. (2014). Antifungal Drug Resistance Evoked *via* RNAi-Dependent Epimutations. Nature 513, 555–558. doi: 10.1038/nature13575 25079329 PMC4177005

[B11] Caramalho R. Tyndall J. D. A. Monk B. C. Larentis T. Lass-Flörl C. Lackner M. (2017). Intrinsic Short-Tailed Azole Resistance in Mucormycetes is Due to an Evolutionary Conserved Aminoacid Substitution of the Lanosterol 14α-Demethylase. Sci. Rep. 7, 15898. doi: 10.1038/s41598-017-16123-9 29162893 PMC5698289

[B12] Chamilos G. Lewis R. E. Kontoyiannis D. P. (2008). Delaying Amphotericin B-Based Frontline Therapy Significantly Increases Mortality Among Patients With Hematologic Malignancy Who Have Zygomycosis. Clin. Infect. Dis. 47, 503–509. doi: 10.1086/590004 18611163

[B13] Chang Z. Billmyre R. B. Lee S. C. Heitman J. (2019). Broad Antifungal Resistance Mediated by RNAi-Dependent Epimutation in the Basal Human Fungal Pathogen *Mucor circinelloides* . PloS Genet. 15, e1007957. doi: 10.1371/journal.pgen.1007957 30742617 PMC6386414

[B14] Chang Z. Heitman J. (2019). Drug-Resistant Epimutants Exhibit Organ-Specific Stability and Induction During Murine Infections Caused by the Human Fungal Pathogen *Mucor circinelloides* . MBio 10, e02579–e02519. doi: 10.1128/mBio.02579-19 31690679 PMC6831780

[B15] Chau A. S. Chen G. McNicholas P. M. Mann P. A. (2006). Molecular Basis for Enhanced Activity of Posaconazole Against *Absidia corymbifera* and *Rhizopus oryzae* . Antimicrob. Agents Chemother. 50, 3917–3919. doi: 10.1128/AAC.00747-06 16966400 PMC1635193

[B16] Chibucos M. C. Soliman S. Gebremariam T. Lee H. Daugherty S. Orvis J.. (2016). An Integrated Genomic and Transcriptomic Survey of Mucormycosis-Causing Fungi. Nat. Commun. 7, 12218. doi: 10.1038/ncomms12218 27447865 PMC4961843

[B17] Cornely O. A. Alastruey-Izquierdo A. Arenz D. Chen S. C. A. Dannaoui E. Hochhegger B.. (2019). Global Guideline for the Diagnosis and Management of Mucormycosis: An Initiative of the European Confederation of Medical Mycology in Cooperation With the Mycoses Study Group Education and Research Consortium. Lancet Infect. Dis. 19, e405–e421. doi: 10.1016/S1473-3099(19)30312-3 31699664 PMC8559573

[B18] Cowen L. E. Sanglard D. Howard S. J. Rogers P. D. Perlin D. S. (2015). Mechanisms of Antifungal Drug Resistance. Cold Spring Harb. Perspect. Med. 5, a019752. doi: 10.1101/cshperspect.a019752 PMC4484955 25384768

[B19] Ganbaatar U. Liu C. (2021). CRISPR-Based COVID-19 Testing: Toward Next-Generation Point-of-Care Diagnostics. Front. Cell. Infect. Microbiol. 11, 373. doi: 10.3389/FCIMB.2021.663949/BIBTEX PMC8120276 33996635

[B20] Gebremariam T. Alkhazraji S. Soliman S. S. M. Gu Y. Jeon H. H. Zhang L.. (2019). Anti-CotH3 Antibodies Protect Mice From Mucormycosis by Prevention of Invasion and Augmenting Opsonophagocytosis. Sci. Adv. 5, eaaw1327. doi: 10.1126/sciadv.aaw1327 31206021 PMC6561750

[B21] Gebremariam T. Liu M. Luo G. Bruno V. Phan Q. T. Waring A. J.. (2014). CotH3 Mediates Fungal Invasion of Host Cells During Mucormycosis. J. Clin. Invest. 124, 237–250. doi: 10.1172/JCI71349 24355926 PMC3871245

[B22] Ghosh A. Sarkar A. Paul P. Patel P. (2021). The Rise in Cases of Mucormycosis, Candidiasis and Aspergillosis Amidst COVID19. Fungal Biol. Rev. 38, 67–91. doi: 10.1016/j.fbr.2021.09.003 34548877 PMC8445778

[B23] Gomes M. Z. R. Lewis R. E. Kontoyiannis D. P. (2011). Mucormycosis Caused by Unusual Mucormycetes, non-*Rhizopus*, -*Mucor*, and -*Lichtheimia* Species. Clin. Microbiol. Rev. 24, 411–445. doi: 10.1128/CMR.00056-10 21482731 PMC3122490

[B24] Gryganskyi A. P. Golan J. Dolatabadi S. Mondo S. Robb S. Idnurm A.. (2018). Phylogenetic and Phylogenomic Definition of Rhizopus Species. G3 8, 2007–2018. doi: 10.1534/g3.118.200235 29674435 PMC5982828

[B25] Hassan M. I. A. Keller M. Hillger M. Binder U. Reuter S. Herold K.. (2021). The Impact of Episporic Modification of *Lichtheimia corymbifera* on Virulence and Interaction With Phagocytes. Comput. Struct. Biotechnol. J. 19, 880–896. doi: 10.1016/j.csbj.2021.01.023 33598103 PMC7851798

[B26] Hussain S. Riad A. Singh A. Klugarová J. Antony B. Banna H.. (2021). Global Prevalence of COVID-19-Associated Mucormycosis (CAM): Living Systematic Review and Meta-Analysis. J. Fungi 7, 985. doi: 10.3390/JOF7110985/S1 PMC8624337 34829271

[B27] Ibragimova S. Szebenyi C. Sinka R. Alzyoud E. I. Homa M. Vágvölgyi C.. (2020). CRISPR-Cas9-Based Mutagenesis of the Mucormycosis-Causing Fungus *Lichtheimia corymbifera* . Int. J. Mol. Sci. 21, 1–11. doi: 10.3390/ijms21103727 PMC7279233 32466287

[B28] Ibrahim A. S. Gebremariam T. Lin L. Luo G. Husseiny M. I. Skory C. D.. (2010). The High Affinity Iron Permease is a Key Virulence Factor Required for *Rhizopus oryzae* Pathogenesis. Mol. Microbiol. 77, 587–604. doi: 10.1111/j.1365-2958.2010.07234.x 20545847 PMC2909342

[B29] Jacobsen I. D. (2019). Animal Models to Study Mucormycosis. J. Fungi 5, 27. doi: 10.3390/jof5020027 PMC6617025 30934788

[B30] Jeong W. Keighley C. Wolfe R. Lee W. L. Slavin M. A. Chen S. C. A.. (2019a). Contemporary Management and Clinical Outcomes of Mucormycosis: A Systematic Review and Meta-Analysis of Case Reports. Int. J. Antimicrob. Agents 53, 589–597. doi: 10.1016/j.ijantimicag.2019.01.002 30639526

[B31] Jeong W. Keighley C. Wolfe R. Lee W. L. Slavin M. A. Kong D. C. M.. (2019b). The Epidemiology and Clinical Manifestations of Mucormycosis: A Systematic Review and Meta-Analysis of Case Reports. Clin. Microbiol. Infect. 25, 26–34. doi: 10.1016/J.CMI.2018.07.011 30036666

[B32] Koshy S. Ismail N. Astudillo C. L. Haeger C. M. Aloum O. Acharige M. T.. (2017). Breath-Based Diagnosis of Invasive Mucormycosis (Im). Open Forum Infect. Dis. 4, S53–S54. doi: 10.1093/OFID/OFX162.124

[B33] Lackner N. Posch W. Lass-Flörl C. (2021). Microbiological and Molecular Diagnosis of Mucormycosis: From Old to New. Microorganisms 9, 1518. doi: 10.3390/microorganisms9071518 34361953 PMC8304313

[B34] Lamoth F. Lewis R. E. Kontoyiannis D. P. (2021). Role and Interpretation of Antifungal Susceptibility Testing for the Management of Invasive Fungal Infections. J. Fungi 7, 17. doi: 10.3390/jof7010017 PMC7823995 33396870

[B35] Lamoth F. Lewis R. E. Kontoyiannis D. P. (2022). Investigational Antifungal Agents for Invasive Mycoses: A Clinical Perspective. Clin. Infect. Dis. 5, 27. doi: 10.1093/cid/ciab1070 34986246

[B36] Lax C. Navarro-Mendoza M. I. Pérez-Arques C. Navarro E. Nicolás F. E. Garre V. (2021). Stable and Reproducible Homologous Recombination Enables CRISPR-Based Engineering in the Fungus *Rhizopus microsporus* . Cell Rep. Methods 1, 100124. doi: 10.1016/j.crmeth.2021.100124 PMC9017206 35475217

[B37] Lee S. C. Li A. Calo S. Inoue M. Tonthat N. K. Bain J. M.. (2015). Calcineurin Orchestrates Dimorphic Transitions, Antifungal Drug Responses and Host-Pathogen Interactions of the Pathogenic Mucoralean Fungus *Mucor circinelloides* . Mol. Microbiol. 97, 844–865. doi: 10.1111/mmi.13071 26010100 PMC4633022

[B38] Li C. H. Cervantes M. Springer D. J. Boekhout T. Ruiz-Vazquez R. M. Torres-Martinez S. R.. (2011). Sporangiospore Size Dimorphism is Linked to Virulence of *Mucor circinelloides* . PloS Pathog. 7, e1002086. doi: 10.1371/journal.ppat.1002086 21698218 PMC3116813

[B39] López-Muñoz A. Nicolás F. E. García-Moreno D. Pérez-Oliva A. B. Navarro-Mendoza M. I. Hernández-Oñate M. A.. (2018). An Adult Zebrafish Model Reveals That Mucormycosis Induces Apoptosis of Infected Macrophages. Sci. Rep. 8, 12802. doi: 10.1038/s41598-018-30754-6 30143654 PMC6109148

[B40] Ma L. J. Ibrahim A. S. Skory C. Grabherr M. G. Burger G. Butler M.. (2009). Genomic Analysis of the Basal Lineage Fungus *Rhizopus oryzae* Reveals a Whole-Genome Duplication. PloS Genet. 5, e1000549. doi: 10.1371/journal.pgen.1000549 19578406 PMC2699053

[B41] Millon L. Herbrecht R. Grenouillet F. Morio F. Alanio A. Letscher-Bru V.. (2016). Early Diagnosis and Monitoring of Mucormycosis by Detection of Circulating DNA in Serum: Retrospective Analysis of 44 Cases Collected Through the French Surveillance Network of Invasive Fungal Infections (RESSIF). Clin. Microbiol. Infect. 22, 810.e1–810.e8. doi: 10.1016/j.cmi.2015.12.006 26706615

[B42] Morio F. Lombardi L. Butler G. (2020). The CRISPR Toolbox in Medical Mycology: State of the Art and Perspectives. PloS Pathog. 16, e1008201. doi: 10.1371/journal.ppat.1008201 31945142 PMC6964833

[B43] Nagy G. Kiss S. Varghese R. Bauer K. Szebenyi C. Kocsubé S.. (2021). Characterization of Three Pleiotropic Drug Resistance Transporter Genes and Their Participation in the Azole Resistance of *Mucor circinelloides* . Front. Cell. Infect. Microbiol. 11, 660347. doi: 10.3389/fcimb.2021.660347 33937100 PMC8079984

[B44] Nagy G. Szebenyi C. Csernetics Á. Vaz A. G. Tóth E. J. Vágvölgyi C.. (2017). Development of a Plasmid Free CRISPR-Cas9 System for the Genetic Modification of *Mucor circinelloides* . Sci. Rep. 7, 16800. doi: 10.1038/s41598-017-17118-2 29196656 PMC5711797

[B45] Navarro-Mendoza M. I. Pérez-Arques C. Murcia L. Martínez-García P. Lax C. Sanchis M.. (2018). Components of a New Gene Family of Ferroxidases Involved in Virulence are Functionally Specialized in Fungal Dimorphism. Sci. Rep. 8, 7660. doi: 10.1038/s41598-018-26051-x 29769603 PMC5955967

[B46] Navarro-Mendoza M. I. Pérez-Arques C. Panchal S. Nicolás F. E. Mondo S. J. Ganguly P.. (2019). Early Diverging Fungus *Mucor circinelloides* Lacks Centromeric Histone CENP-A and Displays a Mosaic of Point and Regional Centromeres. Curr. Biol. 29, 3791–3802.e6. doi: 10.1016/j.cub.2019.09.024 31679929 PMC6925572

[B47] Nguyen M. H. Kaul D. Muto C. Cheng S. J. Richter R. A. Bruno V. M.. (2020). Genetic Diversity of Clinical and Environmental Mucorales Isolates Obtained From an Investigation of Mucormycosis Cases Among Solid Organ Transplant Recipients. Microb. Genomics 6, e000473. doi: 10.1099/MGEN.0.000473 PMC8116672 33245689

[B48] Nicolás F. E. Navarro-Mendoza M. I. Pérez-Arques C. López-García S. Navarro E. Torres-Martínez S.. (2018). “Molecular Tools for Carotenogenesis Analysis in the Mucoral *Mucor circinelloides* ,” in Methods in Molecular Biology (New York, NY: Humana Press), 221–237. doi: 10.1007/978-1-4939-8742-9_13 30109634

[B49] Patiño-Medina J. A. Maldonado-Herrera G. Pérez-Arques C. Alejandre-Castañeda V. Reyes-Mares N. Y. Valle-Maldonado M. I.. (2018). Control of Morphology and Virulence by ADP-Ribosylation Factors (Arf) in *Mucor circinelloides* . Curr. Genet. 64, 853–869. doi: 10.1007/s00294-017-0798-0 29264641

[B50] Patiño-Medina J. A. Reyes-Mares N. Y. Valle-Maldonado M. I. Jácome-Galarza I. E. Pérez-Arques C. Nuñez-Anita R. E.. (2019a). Heterotrimeric G-Alpha Subunits Gpa11 and Gpa12 Define a Transduction Pathway That Control Spore Size and Virulence in *Mucor circinelloides* . PloS One 14, e0226682. doi: 10.1371/journal.pone.0226682 31887194 PMC6936849

[B51] Patiño-Medina J. A. Valle-Maldonado M. I. Maldonado-Herrera G. Pérez-Arques C. Jácome-Galarza I. E. Díaz-Pérez C.. (2019b). Role of Arf-Like Proteins (Arl1 and Arl2) of *Mucor circinelloides* in Virulence and Antifungal Susceptibility. Fungal Genet. Biol. 129, 40–51. doi: 10.1016/j.fgb.2019.04.011 31014992

[B52] Pérez-Arques C. Navarro-Mendoza M. I. Murcia L. Lax C. Martínez-García P. Heitman J.. (2019). *Mucor circinelloides* Thrives Inside the Phagosome Through an Atf-Mediated Germination Pathway. MBio 10, e02765–e02718. doi: 10.1128/mBio.02765-18 30723131 PMC6428757

[B53] Pérez-Arques C. Navarro-Mendoza M. I. Murcia L. Navarro E. Garre V. Nicolás F. E. (2020). A non-Canonical RNAi Pathway Controls Virulence and Genome Stability in Mucorales. PloS Genet. 16, e1008611. doi: 10.1371/journal.pgen.1008611 32658892 PMC7377519

[B54] Prakash H. Chakrabarti A. (2019). Global Epidemiology of Mucormycosis. J. Fungi 5, E26. doi: 10.3390/jof5010026 PMC6462913 30901907

[B55] Revie N. M. Iyer K. R. Robbins N. Cowen L. E. (2018). Antifungal Drug Resistance: Evolution, Mechanisms and Impact. Curr. Opin. Microbiol. 45, 70–76. doi: 10.1016/j.mib.2018.02.005 29547801 PMC6135714

[B56] Rocchi S. Scherer E. Mengoli C. Alanio A. Botterel F. Bougnoux M. E.. (2021). Interlaboratory Evaluation of Mucorales PCR Assays for Testing Serum Specimens: A Study by the Fungal PCR Initiative and the Modimucor Study Group. Med. Mycol. 59, 126–138. doi: 10.1093/mmy/myaa036 32534456

[B57] Roden M. M. Zaoutis T. E. Buchanan W. L. Knudsen T. A. Sarkisova T. A. Schaufele R. L.. (2005). Epidemiology and Outcome of Zygomycosis: A Review of 929 Reported Cases. Clin. Infect. Dis. 41, 634–653. doi: 10.1086/432579 16080086

[B58] Rodríguez-Frómeta R. A. Gutiérrez A. Torres-Martínez S. Garre V. (2013). Malic Enzyme Activity is Not the Only Bottleneck for Lipid Accumulation in the Oleaginous Fungus *Mucor circinelloides* . Appl. Microbiol. Biotechnol. 97, 3063–3072. doi: 10.1007/s00253-012-4432-2 23053085

[B59] Sagatova A. A. Keniya M. V. Wilson R. K. Sabherwal M. Tyndall J. D. A. Monk B. C. (2016). Triazole Resistance Mediated by Mutations of a Conserved Active Site Yyrosine in *Fungal lanosterol* 14α-Demethylase. Sci. Rep 6, 26213. doi: 10.1038/srep26213 27188873 PMC4870556

[B60] Schwarz P. Guedouar H. Laouiti F. Grenouillet F. Dannaoui E. (2019). Identification of Mucorales by Matrix-Assisted Laser Desorption Ionization Time-Of-Flight Mass Spectrometry. J. Fungi 5, 56. doi: 10.3390/JOF5030056 PMC6787577 31269718

[B61] Shi L. Zhao X. Yan X. Liu Y. Liu Y. Cao H.. (2021). Aggressive Disseminated *Rhizomucor pusillus* Infection in a Ph-Like Acute Lymphoblastic Leukemia Patient: Early Detection by Cell-Free DNA Next-Generation Sequencing. J. Infect. Chemother. 28, 459–464. doi: 10.1016/j.jiac.2021.12.007 34955408

[B62] Skiada A. Lass-Floerl C. Klimko N. Ibrahim A. Roilides E. Petrikkos G. (2018). Challenges in the Diagnosis and Treatment of Mucormycosis. Med. Mycol. 56, S93–S101. doi: 10.1093/mmy/myx101 PMC6251532 29538730

[B63] Skiada A. Pavleas I. Drogari-Apiranthitou M. (2020). Epidemiology and Diagnosis of Mucormycosis: An Update. J. Fungi 6, 265. doi: 10.3390/jof6040265 PMC7711598 33147877

[B64] Soare A. Y. Watkins T. N. Bruno V. M. (2020). Understanding Mucormycoses in the Age of “Omics.” Front. Genet. 11, 699. doi: 10.3389/fgene.2020.00699 32695145 PMC7339291

[B65] Soliman S. S. M. Baldin C. Gu Y. Singh S. Gebremariam T. Swidergall M.. (2021). Mucoricin is a Ricin-Like Toxin That is Critical for the Pathogenesis of Mucormycosis. Nat. Microbiol. 6, 313–326. doi: 10.1038/s41564-020-00837-0 33462434 PMC7914224

[B66] Stone N. Gupta N. Schwartz I. (2021). Mucormycosis: Time to Address This Deadly Fungal Infection. Lancet Microbe 2, e343–e344. doi: 10.1016/S2666-5247(21)00148-8 35544192

[B67] Valle-Maldonado M. I. Patiño-Medina J. A. Pérez-Arques C. Reyes-Mares N. Y. Jácome-Galarza I. E. Ortíz-Alvarado R.. (2020). The Heterotrimeric G-Protein Beta Subunit Gpb1 Controls Hyphal Growth Under Low Oxygen Conditions Through the Protein Kinase A Pathway and is Essential for Virulence in the Fungus *Mucor circinelloides* . Cell. Microbiol. 22, e13236. doi: 10.1111/cmi.13236 32562333

[B68] Vellanki S. Billmyre R. B. Lorenzen A. Campbell M. Turner B. Huh E. Y.. (2020). A Novel Resistance Pathway for Calcineurin Inhibitors in the Human-Pathogenic Mucorales *Mucor circinelloides* . MBio 11, e02949-19. doi: 10.1128/mBio.02949-19 31992620 PMC6989107

[B69] Vellanki S. Navarro-Mendoza M. I. Garcia A. B. Murcia L. Perez-Arques C. Garre V.. (2018). *Mucor circinelloides*: Growth, Maintenance and Genetic Manipulation. Curr. Protoc. Microbiol. 49, e53. doi: 10.1002/jbmr.548.Cell 30040216 PMC6060640

[B70] Waldorf A. R. Levitz S. M. Diamond R. D. (1984). *In Vivo* Bronchoalveolar Macrophage Defense Against *Rhizopus oryzae* and *Aspergillus fumigatus* . J. Infect. Dis. 150, 752–760. doi: 10.1093/infdis/150.5.752 6387001

[B71] Walther G. Wagner L. Kurzai O. (2019). Updates on the Taxonomy of Mucorales With an Emphasis on Clinically Important Taxa. J. Fungi 5, 106. doi: 10.3390/jof5040106 PMC6958464 31739583

[B72] Walther G. Wagner L. Kurzai O. (2020). Outbreaks of Mucorales and the Species Involved. Mycopathologia 185, 765–781. doi: 10.1007/s11046-019-00403-1 31734800

[B73] Watkins T. N. Gebremariam T. Swidergall M. Shetty A. C. Graf K. T. Alqarihi A.. (2018). Inhibition of EGFR Signaling Protects From Mucormycosis. MBio 9, e01384-18. doi: 10.1128/mBio.01384-18 30108171 PMC6094478

[B74] Wurster S. Ruiz O. E. Samms K. M. Tatara A. M. Albert N. D. Kahan P. H.. (2021). EGF-Mediated Suppression of Cell Extrusion During Mucosal Damage Attenuates Opportunistic Fungal Invasion. Cell Rep. 34, 108896. doi: 10.1016/j.celrep.2021.108896 33761358 PMC8842569

[B75] Wurster S. Tatara A. M. Albert N. D. Ibrahim A. S. Heitman J. Lee S. C.. (2020). Tornadic Shear Stress Induces a Transient, Calcineurin-Dependent Hypervirulent Phenotype in Mucorales Molds. MBio 11, 1–15. doi: 10.1128/mBio.01414-20 PMC7327176 32605990

